# Threat Assessment of Buried Objects Using Single-Frequency Microwave Measurements

**DOI:** 10.3390/s25165132

**Published:** 2025-08-19

**Authors:** İbrahim Halil Bayat, Gülçin Yarimay, Semih Doğu, İbrahim Akduman

**Affiliations:** 1 Electronics and Communication Engineering Department, Istanbul Technical University, 34469 Istanbul, Turkey; yarimay@itu.edu.tr (G.Y.); dogu16@itu.edu.tr (S.D.); akduman@itu.edu.tr (İ.A.); 2 Mechanical and Chemical Industry Corporation, 06560 Ankara, Turkey

**Keywords:** microwave systems, neural network, buried object detection

## Abstract

This study presents a lightweight neural network model integrated with a microwave-based detection system for identifying buried objects. The proposed model is trained and tested exclusively on real-world measurements, enhancing its practical relevance and robustness. The system utilizes 16 × 16 scattering parameter (S-parameter) measurements, transformed into a compact 256-dimensional feature vector that captures the microwave response of subsurface materials. This representation enables a neural network architecture with reduced computational complexity while maintaining high accuracy. Experimental evaluations demonstrate that the proposed model achieves an accuracy of 99.83%, an F1 score of 0.989, and a recall of 0.979 in distinguishing hazardous from non-hazardous (safe) objects, outperforming baseline CNN, DRN, and EfficientNet architectures. These results confirm the suitability of the approach in defense and security applications.

## 1. Introduction

Detecting buried objects holds critical importance due to its impacts on public safety, military strategy, and humanitarian operations [1]. In regions affected by ongoing armed conflict, the presence of improvised explosive devices constitutes a persistent and often invisible threat. These concealed explosive hazards jeopardize not only the lives of civilians, particularly in post-conflict zones where routine activities like farming, construction, or travel can inadvertently trigger deadly devices [2], but also the safety and operational effectiveness of military personnel deployed in hostile or unstable environments [3]. Beyond the immediate physical harm, such explosives contribute to long-term psychological trauma [4].

The primary concern within military and defense contexts is not merely detecting buried objects but accurately assessing whether they pose a threat. Timely identifying hazardous items such as explosives is critical for safeguarding personnel, preserving mission integrity, and ensuring operational success. In real time, the ability to neutralize these hidden threats enhances situational awareness, reduces the risk of ambushes or sabotage, and allows for the secure movement of personnel and equipment across contested terrains. Microwave technology offers a promising solution to the pressing need for effective detection techniques, primarily because electromagnetic waves exhibit favorable propagation characteristics in the microwave frequency range [5].

Unlike optical, infrared, or acoustic systems that may suffer from limited penetration in lossy environments, microwave signals are capable of penetrating a wide range of non-metallic and heterogeneous materials such as soil, sand, and rubble, making them well-suited for subsurface sensing applications [6]. This ability to probe beneath the surface without physical excavation provides a distinct advantage for detecting deliberately hidden threats. In buried object detection, the fundamental principle involves the emission of microwave signals toward the ground and the subsequent collection and interpretation of the backscattered responses. The variations in these scattered signals—caused by discontinuities in permittivity or conductivity—can be analyzed to infer the location, shape, size, depth, and potentially even the material composition of subsurface anomalies, including explosive devices. Over the past decade, the application of microwave-based techniques has expanded well beyond the domain of subsurface detection [7,8,9,10].

Despite the many advantages and advancements associated with microwave detection systems, several technical and practical challenges still affect their performance and widespread deployment. One significant limitation lies in the inherently weak contrast in electrical properties between certain target materials and their surrounding environment, which can lead to ambiguous or missed detections [11,12]. Additionally, environmental noise, signal attenuation, and clutter, such as rocks and roots, can introduce uncertainties and degrade image quality [13]. The complexity is further compounded by multiple scattering effects and signal diffraction, which can obscure or distort the signals reflected from the buried targets.

In response to these challenges, integrating artificial intelligence (AI), particularly machine learning and deep learning algorithms, has gained significant importance over the past decade [14]. AI-based approaches are increasingly used to enhance signal interpretation, automate object recognition, and improve the robustness of detection systems under diverse and uncertain environmental conditions [15,16,17]. By training models on large datasets of microwave signatures, these systems can learn to distinguish between true threats and benign anomalies, significantly reducing false positives and improving overall detection accuracy.

## 2. Background and Related Work

One study utilized finite-difference time-domain-based simulations to estimate geophysical parameters such as object radius and lateral and vertical positions. The study converted two-dimensional B-Scan images into one-dimensional data. The study also compared the performance of support vector machines (SVMs) and convolutional neural networks (CNNs), achieving an absolute error of approximately 10 mm, or 8% at a center frequency of 480 MHz, with preprocessing steps including background removal and air–ground interface correction [18]. However, a key limitation of this approach is the high computational cost associated with the processing steps of B-Scan data, and the deep architecture of the network, which may hinder real-time deployment. Building upon this, the same group proposed a more advanced three-dimensional simulation model, replacing dense layers with convolutional structures to better capture spatial patterns of buried objects, again focusing on estimating depth, position, and radius [19]. Nonetheless, the proposed DRN model was trained only on cylindrical PEC (perfect electric conductor) objects, and its ability to generalize to different material types and shapes remains limited, especially in more complex subsurface environments.

In another approach, a multi-step pipeline was implemented for object detection, involving an initial segmentation stage to isolate regions of interest, followed by a sliding window method for localized analysis. A two-part data augmentation process then supplemented this to enhance the model’s performance across varied scenarios. The comprehensive nature of this pipeline contributed to improved performance. Initially, SVM outperformed CNN with an accuracy of 86.9%, but after the application of data augmentation, CNN surpassed SVM with an impressive 96% accuracy [20]. However, the method heavily relies on data preprocessing and augmentation steps, and CNN performance may degrade in data-scarce environments. Moreover, the proposed system has not been validated using real-world measurements, which limits its practical applicability.

Object classification has also been addressed through CNN-based models trained on large ground penetrating radar (GPR) datasets. A binary classifier trained on 1080 GPR images could distinguish between metallic and non-metallic objects, such as stones, cans, bottles, and nails, with near-perfect accuracy [21]. However, the dataset used in this study had limited diversity and consisted of clean, controlled data, which may not reflect the complexity of real-world conditions and could limit the model’s generalizability.

The size estimation of buried objects has been tackled using edge-detection filters for feature extraction and CNNs trained on B-Scan images. The model achieved a mean absolute error (MAE) of 6.74 mm on previously unseen data, demonstrating strong generalization performance [22]. However, the model was mostly trained on metallic cylindrical objects, which may limit its performance and generalization when applied to other object types or shapes. Additionally, the model has not been validated using real GPR data, raising questions about its effectiveness in practical scenarios.

Electromagnetic imaging has also been explored using deep convolutional neural networks (DCNNs) for the reconstruction of the two-dimensional (2D) shape of buried conductors [23]. In this approach, a DCNN model was designed to map scattered field data into accurate 2D representations of subsurface objects. The model demonstrated promising results, achieving a reconstruction loss as low as 2.95%, which marked a notable improvement over baseline models such as U-Net, a widely used architecture in inverse imaging problems. By leveraging the hierarchical feature extraction capability of DCNNs, the method effectively captured the spatial distribution of electromagnetic responses and translated them into meaningful geometric reconstructions of the buried targets. This performance suggests that DCNNs can serve as powerful tools in addressing inverse scattering problems within the domain of buried object detection. However, the model’s current application is limited by its reliance solely on synthetic data, as it has not been validated on real-world measurements. This lack of empirical testing raises concerns about the model’s robustness and generalization capability when exposed to measurement noise, soil heterogeneity, and other practical complexities commonly encountered in field scenarios.

Another notable approach focuses on enhancing the speed and efficiency of buried object characterization by eliminating the need for extensive preprocessing [24]. In this study, a time–frequency regression model (FRM) was employed to directly process raw ground-penetrating radar (GPR) signals, thereby accelerating the data interpretation pipeline and reducing latency in detection systems. By leveraging data-driven surrogate models, the method efficiently estimated key object parameters without requiring intermediate signal conditioning stages, such as background subtraction or feature extraction. This streamlined process contributed to improved velocity and computational speed, showcasing the practical advantages of integrating surrogate modeling with GPR-based detection frameworks. However, a key limitation of this method lies in its validation process—it was tested solely on a single, cylindrical perfect electric conductor (PEC) object. This narrow validation scope raises concerns about the model’s generalizability to real-world conditions, where buried objects may vary widely in shape, material composition, and electromagnetic response.

A recent approach to buried object detection has focused on the direct classification of A-scan images using deep learning models [25]. In this method, simulated ground penetrating radar (GPR) reflection signals were transformed into image representations, which were then used as input for neural networks designed to distinguish between different types of buried objects. The study explored various architectures, including EfficientNet-b0, ResNet-18, Inception-ResNet-v2, and ResNet-101, all of which achieved a maximum validation accuracy of 87.5% under controlled conditions. The classification task relied on a relatively small dataset of 60 images, equally divided among several object categories including metallic, plastic, and pottery materials. While the results demonstrate that deep networks can capture meaningful patterns from reflection signals, the limited dataset poses a significant constraint on the model’s generalizability. Moreover, because the models were trained exclusively on simulated data, their robustness in the presence of real-world noise or variations in burial conditions remains unverified. Consequently, the practical applicability of this method in field conditions is yet to be fully established.

Another recent study introduced a novel deep learning framework utilizing Generative Adversarial Networks (GANs) for electromagnetic imaging of buried objects [26]. The approach begins with a Born-based pre-scatterer (BPS) method to generate coarse initial reconstructions, which are subsequently refined using a GAN. In this architecture, the generator network is trained to produce images that closely resemble the true electromagnetic response, while the discriminator aims to distinguish between real and generated data, thereby improving reconstruction fidelity through adversarial learning. The proposed method was evaluated on synthetic data and demonstrated superior performance compared to traditional U-Net architectures, particularly in scenarios involving buried objects with varied dielectric properties and irregular shapes. However, despite these promising results, several limitations remain. The reliance on the BPS step introduces a preprocessing dependency that may affect the generalizability of the approach. Additionally, GANs are known for their training instability and sensitivity to the design of loss functions, which requires careful tuning to achieve convergence. Most notably, the model’s effectiveness has only been demonstrated using simulated data, and its performance on real-world GPR measurements remains unverified, leaving its practical applicability uncertain.

Beyond usage of GPR, thermal imaging has also been explored as a method for detecting buried objects and landmines through the use of deep learning techniques, specifically region-based convolutional neural networks (RCNN) [27]. This approach leverages temperature variations between buried objects and the surrounding soil to identify both metallic and non-metallic threats, offering an alternative modality to traditional electromagnetic methods. By analyzing thermal images, the model can highlight subtle thermal signatures that correspond to the presence of buried objects, potentially improving detection accuracy in certain scenarios. However, this method faces notable challenges related to environmental conditions. Factors such as soil composition, moisture content, burial depth, and the time of day significantly influence thermal contrast and, consequently, the model’s detection capability.

Lastly, a recent study introduced an innovative classification model for buried objects leveraging second-order deep learning techniques, which enhances the traditional convolutional neural network (CNN) framework by incorporating covariance matrices derived from intermediate convolutional layer outputs [28]. The approach begins by extracting hyperbola thumbnails from ground-penetrating radar (GPR) images, which serve as inputs to the initial layers of a classical CNN. Rather than using the CNN outputs directly for classification, the model computes a covariance matrix representing second-order statistical moments capturing richer structural information of the feature maps. This covariance matrix is then processed through specialized layers designed to classify symmetric positive definite (SPD) matrices, enabling the model to effectively learn complex patterns characteristic of buried objects. Evaluation on a large and diverse dataset demonstrated that this second-order model outperforms both shallow neural networks tailored for GPR data and conventional CNN architectures commonly employed in computer vision, particularly in challenging scenarios involving limited training samples or the presence of mislabeled data. The study further highlighted the model’s robustness when the training and testing data are collected under varying environmental conditions, such as different weather modes, emphasizing its generalization capabilities. Moreover, the authors suggested that this architectural paradigm could extend beyond GPR to other radar-based applications in remote sensing and even to medical fields like EEG analysis, where noisy data and scarce labeled samples are prevalent. However, a notable limitation of this approach is its inherent complexity, which may present challenges in implementation and computational efficiency.

These studies collectively emphasize the value of machine learning algorithms in buried object detection, where architectural choices, preprocessing techniques, data representation, and sensor modality significantly influence model performance and reliability. In this study, a neural network model was developed to enhance the detection and classification of buried objects using data collected from a microwave measurement system. Unlike many previous works relying heavily on simulated data, the proposed model is trained and tested exclusively on real measurement data, ensuring practical relevance and robustness in real-world scenarios. Furthermore, by utilizing scattering (S) parameters as input features, the network architecture remains simple and lightweight compared to traditional deep learning models that process raw or high-dimensional data. The integration of artificial intelligence aims to improve the accuracy of subsurface anomaly detection. The neural network was trained on features extracted from the microwave signals, enabling it to learn complex patterns associated with the presence of explosives beneath the surface. This AI-enhanced approach demonstrates the potential to support real-time decision-making in hazardous environments, offering a promising tool for applications in defense operations. The results indicate that the proposed system effectively distinguishes between hazardous, possible explosive, and non-hazardous buried materials, enabling reliable threat assessment.

## 3. Materials and Methods

### 3.1. Data Gathering

The data used in this study were collected with a fully automated microwave measurement system capable of transmitting, receiving, recording data, and moving the antenna array. The antennas operate below the −10 dB reflection coefficient level at the operating frequency of 700 MHz. This frequency was selected due to the increased penetration capability of electromagnetic waves at lower frequencies compared to the GHz range. The antenna array, which measures 16-port reflection and transmission coefficients and is positioned 30 cm above the ground surface, was moved inside the test area after each acquisition to perform a total of 11 measurements per configuration.

**Figure 1:**
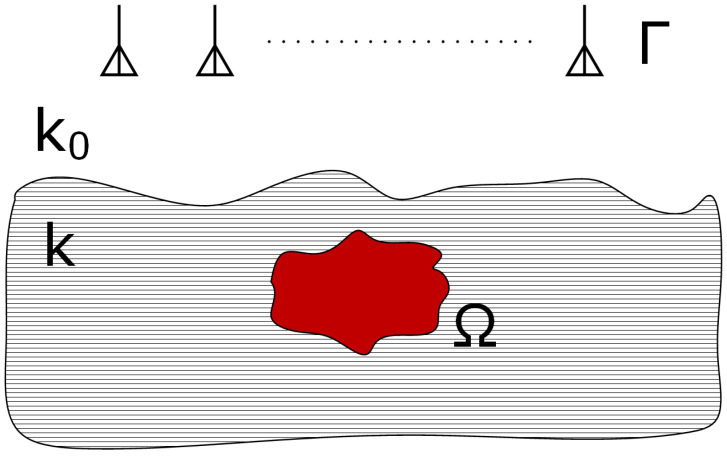
Configuration of the buried object scenario.

A pool was constructed and placed inside an anechoic chamber for the experiments. The pool was filled with gravelly sand, and the chamber temperature was maintained at 20 °C throughout the measurements. The configuration of the buried object problem is depicted in Figure 1. The scattered S-parameters governing the considered problem can be defined as follows [29,30]:

(1) $$\begin{array}{c} S_{a,b}^{\mathrm{sca}}=\int_{\Omega }g\left(m_{a},r^{\prime }\right)\chi \left(r^{\prime }\right)e\left(n_{b},r^{\prime }\right)d\Omega \left(r^{\prime }\right),\;r^{\prime }\in \Omega ;\;m_{a},n_{b}\in \Gamma \end{array}$$

The total S-parameters employed in the experiments can be calculated by $S_{a,b}^{\mathrm{tot}}+S_{a,b}^{\mathrm{sca}}+S_{a,b}^{\mathrm{inc}}$, where $S_{a,b}^{\mathrm{inc}}$ denotes the incident S-parameters. The transmitting and receiving antenna locations are denoted by $n_{b}$ and $m_{a}$, respectively. The object function is given by $\chi \left(r\right)=k\left(r^{\prime }\right)-k_{0}$, where $k\left(r^{\prime }\right)$ and $k_{0}$ denote the wavenumbers of the soil and the background medium, respectively. Green’s function is expressed as follows:

(2) $$\begin{array}{c} g\left(m_{a},r^{\prime }\right)=\frac{iZ_{0}}{2\omega \mu }e^{\mathrm{inc}}\left(m_{a},r^{\prime }\right) \end{array}$$

where $Z_{0}$ is the input impedance of the antenna and ω is the angular frequency. The e term represents the normalized total object field generated by the transmitting antenna, and $e^{\mathrm{inc}}$ is the normalized incident field. For detailed information on these formulations, we refer to [29,30].

**Figure 2:**
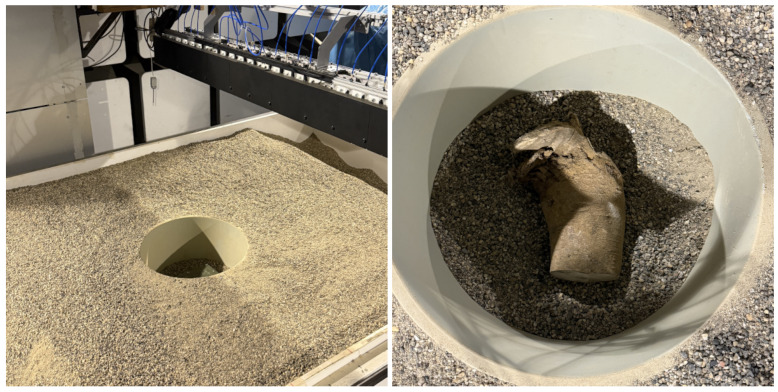
(**Left**) The experimental setup and (**Right**) one sample from the dataset.

The experimental dataset contains 15 distinct object classes, consisting of 4 hazardous (threat) type metals and powders enclosed within specific containers and 11 non-hazardous (safe) types, including wood chips, plastic, and glass cans. Additionally, empty ground measurements (no object present) were also included in the non-hazardous category, serving as control samples. These objects were buried at four different depths (10 cm, 15 cm, 20 cm, and 25 cm) to ensure a diverse and representative dataset for model training and evaluation. In total, 583 measurements were collected. The hazardous objects represent rare, high-risk cases, whereas the non-hazardous category encompasses common background materials. The experimental setup and an example of a buried object are shown in Figure 2.

**Figure 3:**
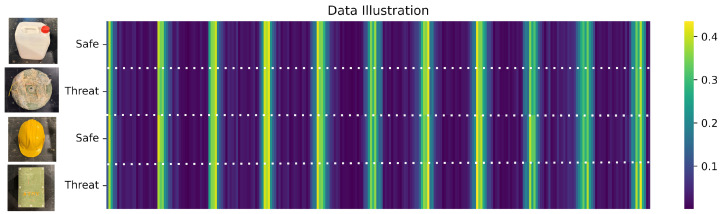
Heatmaps of S-parameter measurements for four samples, illustrating distinct patterns for threat (hazardous) and safe (non-hazardous) buried objects.

To further illustrate the data characteristics, Figure 3 presents heatmaps of scattering parameter (S-parameter) measurements for four selected samples: two labeled as threats and two as safe. These visualizations highlight distinct microwave response patterns captured by the system for each class, demonstrating the potential of S-parameter features for reliable classification of buried threats in complex subsurface environments.

### 3.2. Model and Experiments

Deep learning, a subset of machine learning, has revolutionized artificial intelligence by allowing models to automatically learn hierarchical representations of data [31]. It is based on artificial neural networks, particularly deep neural networks (DNNs) with multiple layers, which can extract complex patterns and features from large datasets [32]. Unlike traditional machine learning approaches that rely on hand-crafted features, deep learning models utilize dense layers, also known as fully connected layers, where each neuron is connected to every neuron in the previous and subsequent layers [33]. These dense layers enable high-dimensional feature learning and play a crucial role in tasks such as classification and regression [34]. Through backpropagation and optimization algorithms, such as stochastic gradient descent, deep learning models continuously update their parameters to improve accuracy.

The primary advantage of dense layers is their ability to capture patterns by leveraging a weighted sum of inputs followed by an activation function, such as the rectified linear unit (ReLU), which is f(x)=max(0,x), or Sigmoid function, which is $\sigma \left(x\right)=1/\left(1+e^{-x}\right)$, to introduce non-linearity [35]. This non-linearity enables DNNs to learn hierarchical feature representations, improving their generalization capabilities across various domains. In this study, the ReLU activation function is utilized throughout the network. In contrast, the Sigmoid function is used in the last layer since the problem has been approached as a classification problem.

**Figure 4:**
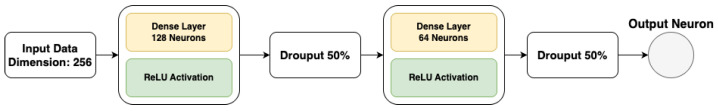
Illustration of neural network.

The fully connected nature of dense layers also introduces specific challenges, including high computational costs and increased trainable parameters, which can lead to overfitting, especially when working with limited data [36]. To mitigate these issues, dropout regularization, batch normalization, and weight decay are commonly employed to improve model generalization and stability [37]. The dropout technique was used for regularization in this work. As ongoing research continues to refine optimization techniques and model architectures, DNNs are expected to advance artificial intelligence and machine learning technologies. Due to this fact, DNNs were used in the network. Figure 4 shows the developed network illustration.

The proposed neural network model comprises a multi-layer perceptron (MLP) architecture with three dense layers, interleaved with dropout layers to mitigate overfitting. The input layer accepts 256-dimensional input vectors. The second layer consists of 128 neurons, followed by a dropout layer. The third dense layer has 64 neurons, which is also followed by a dropout layer before outputting a binary classification result. The model is trained using the Adam optimizer with a default learning rate of 0.001, a batch size of 32, and a dropout rate of 0.5. Binary Cross-Entropy (BCE), was employed as the loss function to guide the learning process and enhance model performance as shown in (3). For inference, the model demonstrates a low computational footprint, with a calculated prediction time of 0.002354 s per sample on a standard CPU and peak memory usage of 102.45 KB. Energy consumption per prediction was between 0.001 and 0.01 joules. These characteristics suggest that the model is well-suited for real-time applications operating under constrained computational and energy resources.

(3) $$BCE=-\frac{1}{N}\sum_{i=1}^{N}y_{i}\log\left(p\left(y_{i}\right)\right)+\left(1-y_{i}\right)\log\left(1-p\left(y_{i}\right)\right)$$

In this study, we used real experimental data measured in the form of S-parameters, which provided a more accurate and realistic representation of the system under analysis compared to synthetic or simulated data. Working with real-world S-parameter data allowed us to capture the complexities and noise inherent in experimental measurements, including errors, environmental influences, and calibration issues. This provided a more authentic dataset that mirrors practical challenges, making the model more robust and adaptable than training solely on idealized synthetic data. Our approach processed the absolute value of S-parameters rather than treating them as separate complex parts. Even though we lose phase information, this choice significantly reduces the complexity of the network and the installation of measurement hardware.

Standardization was applied to the data to ensure that the input features were centered around zero with unit variance. Standardization is a crucial preprocessing step in machine learning, particularly when dealing with numerical data that spans different scales and distributions. Unlike normalization, which scales data to a fixed range, standardization transforms the data by subtracting the mean and dividing by the standard deviation, making it more suitable for deep learning models that rely on gradient-based optimization. Since S-parameters can exhibit significant variations in magnitude across different frequencies and measurements, directly using raw values could lead to biased learning, where larger-magnitude inputs disproportionately influence model training. By standardizing the data, we ensured that each component contributed equally to the learning process, preventing dominance by high-magnitude values and improving numerical stability during optimization. Furthermore, standardization enhances the efficiency of activation functions such as ReLU and sigmoid, which operate more effectively when inputs are distributed around zero.

**Figure 5:**
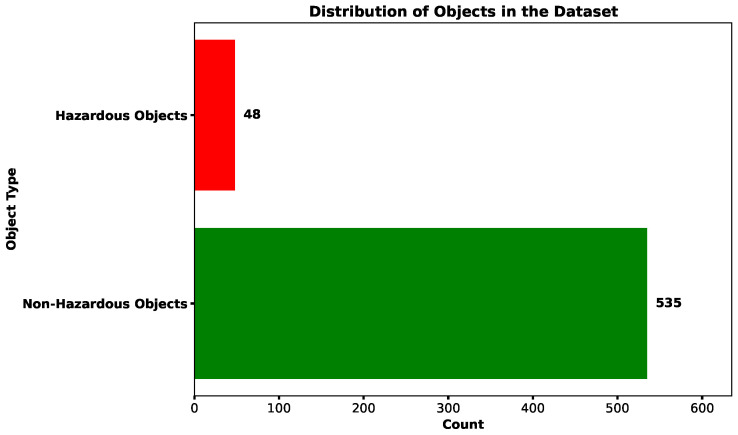
Distribution of hazardous threats in the dataset.

The system consists of 16 antennas, resulting in a measurement dimension of 256 (16 × 16) when each scan is represented as a vector. The dataset comprises a total of 583 samples, of which 48 are labeled as threats, indicating the presence of potentially hazardous materials. The class distribution is illustrated in Figure 5. While the number of threat samples may appear limited, this is both expected and acceptable. In real-world security scenarios, threats represent anomalous or rare events by nature, and, therefore, datasets in this domain inherently exhibit class imbalance. Such imbalance necessitates careful validation strategies to ensure reliable model evaluation.

To comprehensively evaluate the robustness and generalization capability of the proposed model, three distinct validation strategies were employed, with the results summarized in Table 1. In the first approach, the dataset was partitioned into separate training, validation, and test sets containing 408, 87, and 88 samples, respectively. This split provided sufficient data for model learning, hyperparameter tuning, and unbiased performance evaluation. Using this method, the model achieved an accuracy of 0.9886, an F1 score of 0.9231, and a recall of 0.8571 on the held-out test set, demonstrating strong discriminative capability even under class imbalance. For clarity, the definitions of accuracy, recall, and F1 score are given as follows:

(4) $$\mathrm{Accuracy}=\frac{TP+TN}{TP+TN+FP+FN}$$

(5) $$\mathrm{Recall}=\frac{TP}{TP+FN}$$

(6) $$F1\;\mathrm{Score}=2\cdot \frac{\mathrm{Precision}\cdot \mathrm{Recall}}{\mathrm{Precision}+\mathrm{Recall}},\;\mathrm{where}\;\mathrm{Precision}=\frac{TP}{TP+FP}$$

Here, *TP*, *TN*, *FP*, and *FN* denote the number of true positives, true negatives, false positives, and false negatives, respectively. Among these metrics, recall is particularly important in contexts where the cost of false negatives is high, as it measures the model’s sensitivity to the target class by quantifying its ability to correctly identify all relevant positive instances. In high-stakes domains such as medical diagnostics, security screening, and fault detection, achieving a high recall is critical to minimize the risk of overlooking rare but consequential events.

Second, leave-one-out cross-validation (LOOCV) was conducted to evaluate the model’s performance with maximal utilization of the limited data. In this method, each sample in the dataset was used once as a test instance while the remainder served as training data. LOOCV yielded an accuracy of 0.9983, F1-score of 0.9895, and recall of 0.9792, indicating exceptional generalization and stability across individual samples.

Finally, K-fold cross-validation with 5 folds was applied to obtain a balanced view of performance across different partitions of the data. The model was trained and evaluated across five distinct folds, ensuring each sample was used for testing exactly once. The average performance across folds showed an accuracy of 0.99, F1-score of 0.92, and recall of 0.86. These results further confirm the model’s robustness and resilience to variance in training data distribution.

## 4. Results

The proposed model demonstrates strong performance in classifying potential threat scenarios from electromagnetic measurements. The neural network achieves high values across key evaluation metrics (accuracy, F1 score, and recall) under various validation strategies, including independent test sets, leave-one-out cross-validation (LOOCV), and 5-fold cross-validation. In particular, the LOOCV results highlight the robustness and generalization capability of the model, with an accuracy of 0.9983, F1 score of 0.989, and recall of 0.979.

**Figure 6:**
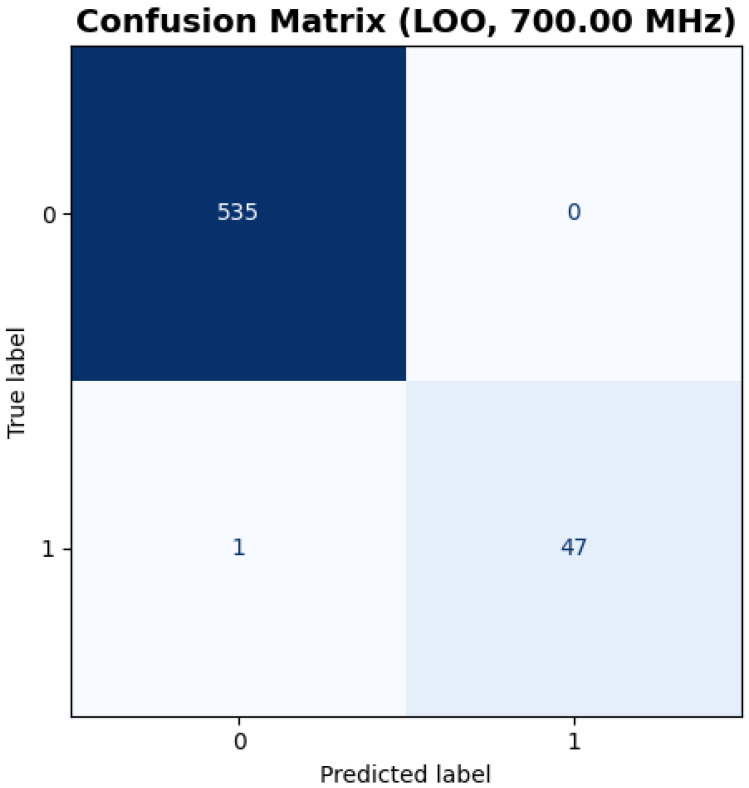
Confusion matrix illustrating the classification results across the entire dataset, highlighting the model’s ability to distinguish between threat and non-threat samples.

Figure 6 presents the confusion matrix derived from the LOOCV evaluation. Out of 583 total samples, only 1 hazardous sample was misclassified as non-hazardous, yielding a near-perfect classification outcome. Specifically, 535 non-threat samples and 47 out of 48 threat samples were correctly identified. This outcome suggests that the model is particularly effective at detecting rare but critical threat events, an essential characteristic in safety-critical applications such as buried explosive detection.

In addition to the overall LOOCV evaluation, experiments were conducted to assess model performance at different burial depths (10 cm, 15 cm, 20 cm, and 25 cm). The results, summarized in Table 2, present accuracy, F1 score, and recall for each depth. The model achieved perfect classification at a 10 cm depth across all metrics. For deeper targets, performance remained consistently high, with accuracy values above 0.99 in all cases and only minor variations in F1 score and recall. The lowest F1 score (0.8696) and recall (0.8333) occurred at 15 cm, while the highest non-perfect performance was observed at 25 cm (accuracy: 0.9979, F1 score: 0.9565, recall: 0.9167). Overall, these results indicate that the proposed approach maintains robust detection capabilities across varying burial depths, with only marginal fluctuations in balanced classification performance.

Despite the relatively small number of threat samples in the dataset, this is a natural consequence of the problem domain. Threat scenarios are inherently rare and constitute anomalous events; hence, a dataset reflective of real-world distributions is expected to be imbalanced. Nevertheless, the model’s ability to correctly classify such minority class instances with minimal false negatives demonstrates its practical applicability and robustness.

Moreover, comparisons with other baseline neural architectures confirm the superior performance of the proposed model, as outlined in Table 3. The results indicate that while the CNN, DRN, and EfficientNet models achieve moderate overall accuracy values of 0.9205, 0.9164, and 0.9013, respectively, their F1 and recall scores are measured as 0.0. This outcome suggests that these architectures failed to correctly identify any positive (threat) samples, thereby producing no true positives in their predictions. In imbalanced classification problems such as the present case, where hazardous instances constitute only a small fraction of the dataset, accuracy alone can be misleading, as a model biased toward predicting only the majority (safe) class can still attain a seemingly acceptable accuracy while entirely neglecting the minority class. The absence of true positives explains the F1 and recall values of zero, given that recall quantifies the proportion of actual threats correctly identified, and the F1 score is the harmonic mean of precision and recall. In contrast, the proposed network demonstrates substantially superior performance across all metrics, achieving an accuracy of 0.9983, an F1 score of 0.9895, and a recall of 0.9792. This indicates that the proposed architecture is capable of not only correctly identifying almost all safe samples but also effectively detecting the rare threat cases, thereby addressing the class imbalance challenge. The significant disparity in performance highlights the critical importance of model design choices, particularly with respect to feature extraction, network depth, and class imbalance handling in ensuring that classification systems for safety-critical applications do not overlook rare but high consequence events.

## 5. Discussion

Although artificial intelligence (AI) applications are often developed for narrowly defined tasks, achieving generalizability across varied conditions remains a fundamental goal for ensuring robustness and real-world applicability. In this study, we addressed the limitations associated with problem-specific modeling by exclusively training the neural network on experimentally acquired data rather than relying on synthetic or simulated datasets.

To further support generalization and mitigate overfitting, we incorporated dropout regularization into the network architecture. This technique, which randomly deactivates a subset of neurons during training, effectively reduces the model’s dependency on any specific subset of features and promotes a more distributed and robust internal representation. Additionally, the input data were simplified through the use of the magnitude-only values of the S-parameters, allowing for a reduction in system cost and better suitability for high frequencies. Relying exclusively on amplitude data can cut measurement equipment costs and improve accuracy due to the presence of uncompensated probe positioning errors in full-data measurements.

The model was, thus, developed with a strong emphasis on key AI design principles, achieving a careful balance between problem specificity and generalization capability. Given the application domain, buried explosive detection, the primary objective was to ensure high sensitivity to hazardous objects, particularly those posing safety risks in defense or humanitarian demining operations. Evaluation on the test set confirmed the effectiveness of the proposed approach, with the model demonstrating high predictive accuracy and consistent classification performance across multiple measurement configurations and burial depths. Looking ahead, future extensions of this work will involve enriching the dataset with a wider variety of explosive materials and container types. This will enable a more thorough evaluation of the model’s generalization capacity and classification resolution across complex scenarios involving diverse threat profiles.

## 6. Conclusions

In this work, a neural network-based approach was successfully integrated with a microwave detection system to enhance the detection of buried explosives. By utilizing 16 × 16 S-parameter measurements to form a 256-dimensional input vector, the model effectively learned to distinguish between hazardous and non-hazardous buried objects. The results demonstrate that AI integration can significantly improve classification accuracy, promising defense application advancement. While the current approach focuses on binary classification of the presence or absence of hazardous objects, future work will extend the framework to support object-wise classification, enabling more detailed identification of object types in the field.

## Figures and Tables

**Table 1:** Performance metrics (accuracy, F1-score, and recall) of the proposed model under three different validation strategies: train/validation/test split, leave-one-out cross-validation (LOOCV), and 5-fold cross-validation.

Validation Type	Accuracy	F1 Score	Recall
Dataset Split	0.988636364	0.923076923	0.857142857
LOOCV	0.998284734	0.989473684	0.979166667
K-Fold (K = 5)	0.994572321	0.928328401	0.865817302

**Table 2:** Classification performance for different burial depths.

Depth (cm)	Accuracy	F1 Score	Recall
10	1.0	1.0	1.0
15	0.993562232	0.869565217	0.833333333
20	0.995708155	0.916666667	0.916666667
25	0.997854077	0.956521739	0.916666667

**Table 3:** Performance comparison of different models on the classification task.

Model	Accuracy	F1 Score	Recall
CNN	0.9205	0.0	0.0
DRN	0.9164	0.0	0.0
EfficientNet	0.9013	0.0	0.0
Proposed Network	0.998284734	0.989473684	0.979166667

## Data Availability

Please direct correspondence and requests for the dataset to G.Y.

## Abbreviations

The following abbreviations are used in this manuscript:

AI	Artificial Intelligence
DL	Deep Learning
LOOCV	Leave-One-Out Cross-Validation
ML	Machine Learning
RELU	Rectified Linear Unit
GPR	Ground Penetrating Radar

## References

[1] Mandal J., Goel M.D., Agarwal A.K. (2021). Surface and buried explosions: An explorative review with recent advances. Arch. Comput. Methods Eng..

[2] Srimuk P., Boonpoonga A., Kaemarungsi K., Athikulwongse K., Dentri S. (2022). Implementation of and experimentation with ground-penetrating radar for real-time automatic detection of buried improvised explosive devices. Sensors.

[3] Rafaels K.A., Gillich P.J., Ehlers R.Z., Duvall P.S. Lower leg injuries in dismounted military personnel from buried explosives. Proceedings of the IRCOBI Conference.

[4] Kislov M.A., Chauhan M., Krupin K.N., Kildyushov E.M., Zotkin D.A. (2022). Forensic pathological characteristics of explosion trauma in confined space terrorist mass fatalities classified with a 3-dimensional model. Leg. Med..

[5] Pastorino M. (2010). Microwave Imaging.

[6] Doğu S., Akıncı M.N., Çayören M., Akduman İ. (2020). Truncated singular value decomposition for through-the-wall microwave imaging application. IET Microwaves Antennas Propag..

[7] Akıncı M.N. (2018). An efficient sampling method for cross-borehole GPR imaging. IEEE Geosci. Remote Sens. Lett..

[8] Zhang X., Tortel H., Ruy S., Litman A. (2011). Microwave imaging of soil water diffusion using the linear sampling method. IEEE Geosci. Remote Sens. Lett..

[9] Counts T., Gurbuz A.C., Scott W.R., McClellan J.H., Kim K. (2007). Multistatic ground-penetrating radar experiments. IEEE Trans. Geosci. Remote Sens..

[10] Paul S., Akhtar M.J. (2023). A novel piecewise Riccati-based SAR microwave imaging technique for the detection of objects inside the layered media. IEEE Trans. Instrum. Meas..

[11] Meaney P.M., Paulsen K.D., Hartov A., Crane R.K. (1995). An active microwave imaging system for reconstruction of 2-D electrical property distributions. IEEE Trans. Biomed. Eng..

[12] Ahmed S.S., Schiessl A., Gumbmann F., Tiebout M., Methfessel S., Schmidt L.-P. (2012). Advanced microwave imaging. IEEE Microw. Mag..

[13] Mohammed B.J., Bialkowski K., Mustafa S., Abbosh A. (2015). Investigation of noise effect on image quality in microwave head imaging systems. IET Microwaves Antennas Propag..

[14] Shao W., Du Y. (2020). Microwave imaging by deep learning network: Feasibility and training method. IEEE Trans. Antennas Propag..

[15] Zardi F., Tosi L., Salucci M., Massa A. (2025). A physics-driven AI approach for microwave imaging of breast tumors. IEEE Trans. Antennas Propag..

[16] Shah P., Moghaddam M. (2017). Super resolution for microwave imaging: A deep learning approach. Proceedings of the 2017 IEEE International Symposium on Antennas and Propagation & USNC/URSI National Radio Science Meeting.

[17] Zhang J., Sharma R., García-Fernández M., Álvarez-Narciandi G., Abbasi M.A.B., Yurduseven O. (2024). Deep learning for sensing matrix prediction in computational microwave imaging with coded-apertures. IEEE Access.

[18] Yurt R., Torpi H., Kizilay A., Koziel S., Pietrenko-Dabrowska A., Mahouti P. (2023). Buried object characterization by data-driven surrogates and regression-enabled hyperbolic signature extraction. Sci. Rep..

[19] Yurt R., Torpi H., Kizilay A., Koziel S., Mahouti P. (2024). Variable data structures and customized deep learning surrogates for computationally efficient and reliable characterization of buried objects. Sci. Rep..

[20] Gharamohammadi A., Shaker G., Crowley M. (2023). Artificial intelligence to detect buried objects. Authorea Prepr..

[21] Sezgin M., Alpdemir M.N. (2023). Classification of buried objects using deep learning on GPR data. Proceedings of the 2023 IEEE International Conference on Advanced Systems and Emergent Technologies (IC_ASET).

[22] Barkataki N., Tiru B., Sarma U. (2022). A CNN model for predicting size of buried objects from GPR B-Scans. J. Appl. Geophys..

[23] Chiu C.-C., Chien W., Yu K.-X., Chen P.-H., Lim E.H. (2023). Electromagnetic imaging for buried conductors using deep convolutional neural networks. Appl. Sci..

[24] Yurt R., Torpi H., Mahouti P., Kizilay A., Koziel S. (2023). Buried object characterization using ground penetrating radar assisted by data-driven surrogate-models. IEEE Access.

[25] Alshamy H.M., Abdul Sadah J.W., Saeed T.R. (2022). Recognizing of the A-Scan Image of a Buried Object Using a Deep Network. Proceedings of the 2nd International Conference on Advances in Engineering Science and Technology (AEST).

[26] Chiu C.-C., Chien W., Li C.-L., Chen P.-H., Yu K.-X., Lim E.H. (2024). Generative Adversarial Network Applied to Electromagnetic Imaging of Buried Objects. Sens. Mater..

[27] Priya C.N.N., Ashok S.D., Maji B., Kumaran K.S. (2021). Deep learning based thermal image processing approach for detection of buried objects and mines. Eng. J..

[28] Jafuno D., Mian A., Ginolhac G., Stelzenmuller N. (2025). Classification of Buried Objects from Ground Penetrating Radar Images by using Second Order Deep Learning Models. IEEE J. Sel. Top. Appl. Earth Obs. Remote Sens..

[29] Haynes M., Moghaddam M. (2011). Vector Green’s Function for S-Parameter Measurements of the Electromagnetic Volume Integral Equation. Proceedings of the 2011 IEEE International Symposium on Antennas and Propagation (APSURSI).

[30] Akinci M.N., Çağlayan T., Özgür S., Alkaşı U., Ahmadzay H., Abbak M., Çayören M., Akduman İ. (2015). Qualitative Microwave Imaging with Scattering Parameters Measurements. IEEE Trans. Microw. Theory Tech..

[31] Janiesch C., Zschech P., Heinrich K. (2021). Machine learning and deep learning. Electron. Mark..

[32] Khan A., Sohail A., Zahoora U., Qureshi A.S. (2020). A survey of the recent architectures of deep convolutional neural networks. Artif. Intell. Rev..

[33] Heaton J. (2018). Ian Goodfellow, Yoshua Bengio, and Aaron Courville: Deep Learning. Genet. Program. Evolvable Mach..

[34] Javid A.M., Das S., Skoglund M., Chatterjee S. (2021). A ReLU dense layer to improve the performance of neural networks. Proceedings of the IEEE International Conference on Acoustics, Speech and Signal Processing (ICASSP).

[35] Waoo A.A., Soni B.K. (2021). Performance analysis of sigmoid and ReLU activation functions in deep neural network. Intelligent Systems: Proceedings of SCIS 2021.

[36] Li H., Li J., Guan X., Liang B., Lai Y., Luo X. (2019). Research on overfitting of deep learning. Proceedings of the 2019 15th International Conference on Computational Intelligence and Security (CIS).

[37] Garbin C., Zhu X., Marques O. (2020). Dropout vs. batch normalization: An empirical study of their impact to deep learning. Multimed. Tools Appl..

